# CMV2b-AGO Interaction Is Required for the Suppression of RDR-Dependent Antiviral Silencing in *Arabidopsis*

**DOI:** 10.3389/fmicb.2016.01329

**Published:** 2016-08-24

**Authors:** Yuan-Yuan Fang, Jian-Hua Zhao, Shang-Wu Liu, Sheng Wang, Cheng-Guo Duan, Hui-Shan Guo

**Affiliations:** ^1^State Key Laboratory of Plant Genomics, Institute of Microbiology, Chinese Academy of SciencesBeijing, China; ^2^Virus-free Seedling Research Institute, Heilongjiang Academy of Agricultural SciencesHarbin, China; ^3^College of Life Sciences, University of Chinese Academy of SciencesBeijing, China

**Keywords:** CMV, viral suppressor, 2b, ARGONAUTE, RDR, RNA silencing

## Abstract

Using a transient plant system, it was previously found that the suppression of *Cucumber mosaic virus* (CMV) 2b protein relies on its double-strand (ds) RNA binding capacity, but it is independent of its interaction with ARGONAUTE (AGO) proteins. Thus, the biological meaning of the 2b-AGO interaction in the context of virus infection remains elusive. In this study, we created infectious clones of CMV mutants that expressed the 2b functional domains of dsRNA or AGO binding and tested the effect of these CMV mutants on viral pathogenicity. We found that the mutant CMV2b_(1–76)_ expressing the 2b dsRNA-binding domain exhibited the same virulence as wild-type CMV in infection with either wild-type Arabidopsis or *rdr1/6* plants with RDR1- and RDR6-deficient mutations. However, remarkably reduced viral RNA levels and increased virus (v)siRNAs were detected in CMV2b_(1–76)_-infected *Arabidopsis* in comparison to CMV infection, which demonstrated that the 2b_(1–76)_ deleted AGO-binding domain failed to suppress the RDR1/RDR6-dependent degradation of viral RNAs. The mutant CMV2b_(8–111)_ expressing mutant 2b, in which the N-terminal 7 amino acid (aa) was deleted, exhibited slightly reduced virulence, but not viral RNA levels, in both wild-type and *rdr1/6* plants, which indicated that 2b retained the AGO-binding activity acquired the counter-RDRs degradation of viral RNAs. The deletion of the N-terminal 7 aa of 2b affected virulence due to the reduced affinity for long dsRNA. The mutant CMV2b_(18–111)_ expressing mutant 2b lacked the N-terminal 17 aa but retained its AGO-binding activity greatly reduced virulence and viral RNA level. Together with the instability of both 2b_(18–111)_-EGFP and RFP-AGO4 proteins when co-expressed in *Nicotiana benthamiana* leaves, our data demonstrates that the effect of 2b-AGO interaction on counter-RDRs antiviral defense required the presence of 2b dsRNA-binding activity. Taken together, our findings demonstrate that the dsRNA-binding activity of the 2b was essential for virulence, whereas the 2b-AGO interaction was necessary for interference with RDR1/6-dependent antiviral silencing in *Arabidopsis*.

## Introduction

RNA silencing (RNA interference, RNAi) is an evolutionarily conserved regulatory mechanism of gene expression in eukaryotes mediated by 20–25-nucleotides (nt) small interference RNAs (siRNAs; Meister and Tuschl, 2004; Baulcombe, 2005). These siRNAs are processed from double-stranded (ds) or hairpin (hp) RNA by Dicer or Dicer-like (DCL) protein. To induce silencing, one strand of a siRNA is loaded into an Argonaute (AGO) protein to form the RNA-induced silencing complex (RISC) and guides the RISC to bind to complementary single-stranded RNA and cleave the RNA. siRNAs-guided AGO-cleaved target RNA may be recognized by RNA-dependent RNA polymerase (RDR), which amplifies the dsRNA substrate for DCLs to produce secondary siRNAs and reinforce the RNA silencing process (Peragine et al., 2004; Axtell et al., 2006; Baulcombe, 2007).

In plants, viral infection also triggers the siRNA-mediated RNA silencing as a natural antiviral defense mechanism. RDR-dependent amplification is a crucial step toward achieving an efficient antiviral defense response in plants. Two of the six *Arabidopsis thaliana* RDRs, RDR1, and RDR6, have been implicated in defense against many viruses, including *Cucumber mosaic virus* (CMV; Dalmay et al., 2000; Qu et al., 2005; Schwach et al., 2005; Vaistij and Jones, 2009; Garcia-Ruiz et al., 2010; Qu, 2010; Ying et al., 2010; Li et al., 2014).

CMV is a tripartite positive-strand RNA virus, which contains three genomic RNAs and two subgenomic RNAs that encode five proteins (Palukaitis and Garcia-Arenal, 2003): two RNA-dependent RNA polymerases, 1a and 2a proteins, and movement protein (MP) encoded by genomic RNA1, RNA2, and RNA3. The 2b protein and the coat protein (CP) are expressed from subgenomic RNA4A and RNA4, which are transcribed from genomic RNA2 and RNA3, respectively (Schwinghamer and Symons, 1975; Ding et al., 1994). The 2b protein expressed from subgenomic RNA4A plays an important role in diverse processes, including symptom induction as a viral virulence determinant, host-specific virus accumulation, the inhibition of RNA silencing and the systemic spread of silencing (Ding et al., 1995; Lucy et al., 2000; Guo and Ding, 2002; Shi et al., 2002). As a viral suppressor of RNA silencing (VSR), the 2b protein has been identified to directly interact with both the long/short dsRNA and AGO proteins (Zhang et al., 2006; Goto et al., 2007; González et al., 2010, 2012; Duan et al., 2012; Hamera et al., 2012), attributed to its complex biochemical and subcellular targeting activity (Duan et al., 2012). In our previous study of the 2b protein encoded by the severe SD isolate from CMV subgroup I, we uncoupled the domain requirements for dsRNA binding and nucleolar targeting from the physical interactions with AGO proteins. We found that dsRNA sequestration is the predominant mechanism by which 2b suppresses silencing and that the 2b-AGO interaction is not essential for its suppressor function. We also found that the direct in *vivo* interactions of the 2b protein with AGO proteins require the functional nucleolar localization signal (NoLS) and redistribute the 2b protein in the nucleus (Duan et al., 2012).

The roles of RNAi-mediated viral immunity against CMV were mostly illustrated using the mutant of CMV that does not express the 2b protein or mutate by amino acid substitution in the N-terminal dsRNA binding domain of the 2b (Diaz-Pendon et al., 2007; Wang et al., 2010; Xu et al., 2013; Dong et al., 2016). These mutants of CMV reduce virulence and virus accumulation in wild-type Arabidopsis plants, but are efficiently rescued in mutant plants defective in RNAi components, such as RDR1, RDR6, or DCL4, which shows that the 2b protein plays critical roles in anti-RNAi defense and that its N-terminal dsRNA binding domain is required for the induction of virulence and virus accumulation in the CMV-infected plants (Diaz-Pendon et al., 2007; Wang et al., 2010; Xu et al., 2013; Dong et al., 2016). We previously found that the 2b-AGO interaction was not essential for the 2b in suppression of silencing, however, we wondered what is the biological significance of the 2b-AGO interaction in the context of virus infection. To this end, we created mutants of CMV from the SD strain that expressed the 2b functional domains of dsRNA- or AGO- binding activity (Duan et al., 2012) and tested the effect of these CMV mutants on viral pathogenicity. We found that the dsRNA-binding activity of the 2b was essential for virulence, whereas the 2b-AGO interaction was necessary for interference with RDR1/6-dependent antiviral silencing in *Arabidopsis*. The possible benefit of the 2b-AGO interaction in CMV infectivity is discussed.

## Materials and methods

### Plant material and growth conditions

*N. benthamiana* plants were grown in a greenhouse at 25°C with 16-h light/8-h dark cycles. *rdr1/6* and wild-type *Arabidopsis* plants were grown in a greenhouse at 22°C with 16-h light/8-h dark cycles.

### Plasmid constructs

35S-R1, 35S-R2, 35S-R3, and 35S-RΔ2b(R2aΔ2b) were described in a previous study (Hou et al., 2011). For R2a2b_(1–76)_, R2a2b_(8–111)_, and R2a2b_(18–111)_ point mutant constructs, mutagenesis was introduced using QuikChange® Lightning Site-Directed Mutagenesis Kits (Agilent Technologies, 210518) according to the manufacturer's instructions. The templates were 35S-R2, 35S-R2, and R2a2b_(8–111)_, and the primer pairs were R2-2b77TGAF/R, R2-2b8-111F/R, and R2-2b18-111F/R, respectively (Table S1).

For the constructs used in dsRNA binding activities, pGEX-4T-2-SD2b was described in a previous study (Duan et al., 2012). The constructs pGEX-4T-2-SD2b_(8–111)_ and pGEX-4T-2-SD2b_(18–111)_ were generated with the pGEX-4T-2-SD2b template using QuikChange® Lightning Site-Directed Mutagenesis Kits (Agilent Technologies, 210518) according to the manufacturer's instructions. The primer pairs were GST2b8-111F/R and GST2b18-111F/R for the constructs pGEX-4T-2-SD2b_(8–111)_ and pGEX-4T-2-SD2b_(18–111)_, respectively (Table S1).

For the constructs used in suppression activity, pBI121-35S-SD2b was described in a previous study (Duan et al., 2012). To generate pBI121-35S-SD2b_(8–111)_, pBI121-35S-SD2b_(18–111)_, and pBI121-35S-SD2b_(1–76)_, the 2b8-111F/2bR, 2b18-111F/2bR, and 2b1-76F/R primers (Table S1) were used to amplify the 2b_(8–111)_/2b_(18–111)_/2b_(1–76)_ mutant fragments with the template of pBI121-35S-SD2b; the resulting fragments were cut by XbaI-SacI and inserted into the XbaI-SacI digested pBI121-35S-SD2b vector to yield pBI121-35S-SD2b_(8–111)_, pBI121-35S-SD2b_(18–111)_, and pBI121-35S-SD2b_(1–76)_.

For constructs used in the subcellular localization and SD2b-AGO colocalization, pBI121-35S-SD2b-EGFP and RFP-AGO4 were described in a previous study (Duan et al., 2012). The constructs pBI121-35S-SD2b_(8–111)_ and pBI121-35S-SD2b_(18–111)_-EGFP were generated with the pBI121-35S-SD2b-EGFP template using QuikChange® Lightning Site-Directed Mutagenesis Kits (Agilent Technologies, 210518) according to the manufacturer's instructions. The primer pairs were 2B8-111-EGFPF/R and 2B18-111-EGFPF/R for the pBI121-35S-SD2b-EGFP and RFP-AGO4 constructs, respectively (Table S1).

### *Agrobacterium tumefaciens*-mediated transient expression and virus inoculation

35S-R1, 35S-R3 and different R2 mutant constructs were co-infiltrated into the leaves of 5-week-old *N. benthamiana* plants, as described in a previous study (Hou et al., 2011). Systemically infected leaves were harvested from pools of 15 to 20 plants for sap extraction for viral infection. Similar levels of viral RNAs in each sap sample estimated by RNA gel blotting were inoculated to *N. benthamiana* and *A. thaliana* seedlings.

### RNA extraction and RNA gel blot analysis

Plant total RNA used for RNA gel blotting was extracted by the hot-phenol method as previously described (Fernández et al., 1997). For the detection of viral RNAs, three 1-kb fragments at the 3′-terminus of each cDNA clone (35S-R1, 35S-R2, and 35S-R3) were amplified, which were then labeled with [a-^32^P] dCTP using the Rediprime II system (GE Healthcare, RPN1633) and were mixed as probes. For the detection of siRNAs, 30 mg of total RNA was separated on 17% polyacrylamide-8 M urea gels. The probes were labeled with [r-^32^P]ATP using T4 PNK (NEB, M0201V). VsiRNAs were detected using mixtures of labeled DNA oligonucleotides specific to RNA3. Signal intensity was quantified using ImageQuant TL software (GE Healthcare).

### Expression and purification of recombinant proteins

For the expression of fusion proteins in *Escherichia coli*, recombinant plasmids were transformed into BL21 cells and induced at 0.3 mM isopropyl b-D-1thiogalactopyranoside (Sigma-Aldrich) in Luria-Bertani medium at 28°C for 3 to 6 h. GST-tagged fusion proteins were purified using Glutathione Sepharose 4B (GE Healthcare, 17-0756-01) according to the manufacturer's instructions.

### EMSAs

21/24-bp ds-siRNA and 55-bp ds-RNA, which were described in a previous study (Duan et al., 2012), were radiolabeled in 50-pmol quantities with 0.3 mM [r-^32^P]ATP and 20 units of T4 PNK (NEB, M0201V). Binding reactions were performed with 1 ng of radiolabeled ds-siRNA and 1 nmol of protein in binding buffer (20 mM Tris-HCl, pH 7.5, 5 mM MgCl2, 300 mM NaCl, 0.1% Nonidet P-40, and protease inhibitor cocktail. After 40 min at room temperature, 1 mL of 50% glycerol and dye was added, and protein RNA complexes were resolved on 6% native polyacrylamide gel. The gels were then exposed to a storage phosphor screen (GE Healthcare).

### Subcellular localization assays

The subcellular localization of 2b mutants and their colocalization with AGO proteins were determined by infiltrating binary plasmids of pBI121-35S-SD2b-EGFP mutants and RFP-AGO4 into 5-week-old *N. benthamiana* leaves, which were maintained for 3 days at 25°C (16-h light/8-h dark). The nuclei were stained with 100 ng/mL 4′,6-diamidino-2-phenylindole (DAPI) for 10 min before confocal microscopy. Confocal fluorescence of GFP, RFP, and DAPI were captured with a Leica SP8 confocal microscope.

## Results

### Construction of the infectious clones of SD-CMV with different mutations in the 2b coding sequence

**Figure 1:**
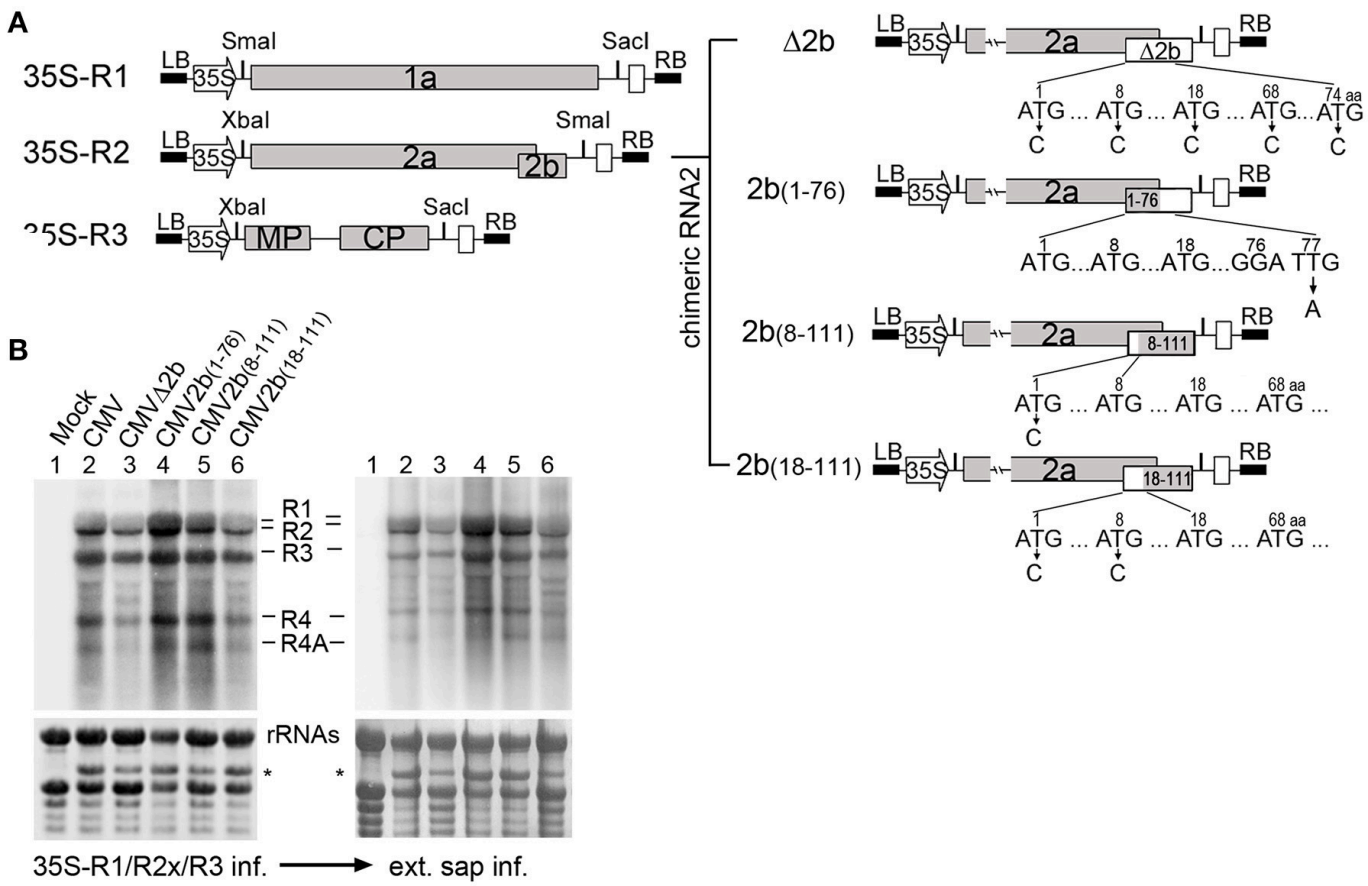
**Construction and biological activities of 2b mutants in the context of CMV infection**. **(A)** Diagram of SD-CMV infectious clone construction. 35S-R1, 35S-R2, and 35S-R3 were the three full-length clones of SD-CMV genomic RNA1, RNA2, and RNA3 as well as four chimeric RNA2 mutants. R2aΔ2b, 2b protein expression was abolished; R2a2b_(1–76)_, with deletion of the C-terminal 35 amino acids; R2a2b_(8–111)_, with deletion of the N-terminal seven amino acids; and R2a2b_(18–111)_, with deletion of the N-terminal 17 amino acids. Substituting “C” for each “T” in the start codon ATG, as well as other ATG codons, or “A” for “T” in creating stop codon present in the 2b coding sequence are indicated with the numbers of the amino acid positions. **(B)** RNA gel blot detection of CMV accumulation in *Agrobacterium*-inoculated leaves (left panel) and the plants inoculated with sap extracted from each CMV2b_(x)_-infected *Nb* leaf (right panel). SD-CMV genomic RNA 3′ UTR was used as a probe. Methylene blue-stained ribosomal rRNA was used as loading control. ^*^A stained viral RNA used as an indicator of SD-CMV infection.

We previously constructed infectious clones of SD-CMV with the viral genomic RNA1, RNA2, and RNA3 under the 35S promoter (Figure 1A) and a chimeric RNA2 (Δ2b) infectious clone, in which 2b protein expression was abolished by nucleotide substitution in the start codon ATG, as well as four other ATG codons in the 2b coding sequence, but 2a protein expression was unaffected, designated as CMVΔ2b (Figure 1A; Hou et al., 2011). To investigate the biological functions of the different biochemical properties of the 2b protein in the context of CMV infection in plants, in this study, we further created mutations of 2b of the genomic RNA2 according to the two main biochemical properties of double-stranded RNA (dsRNA) and AGO binding activities (Duan et al., 2012). As shown in Figure 1A, in addition to the above Δ2b, three 2b mutants were created by deleting the C-terminal 35 amino acids (aa) and creating a stop codon in the 2b coding sequence to yield 2b_(1–76)_ without affecting the overlapping portion of the 2a polymerase. Another two mutants were created by deleting the N-terminal 7 or 17 aa to yield 2b_(8–111)_ and 2b_(18–111)_ by nucleotide substitution in the 1st or both the 1st and 8th “ATG” codons of the 2b coding sequence without affecting the 2a protein. To obtain viral sources of chimeric CMV with different 2b mutant, each of these constructs was transformed into *Agrobacterium* for the infiltration of *N. benthamiana* in the presence of 35S-RNA1 and 35S-RNA3 to examine the infectious properties of the chimeric CMV with 35S-2bx (x represents different 2b mutations shown in Figure 1A), and the related viruses were referred to as wild-type CMV, CMVΔ2b, CMV2b_(1–76)_, CMV2b_(8–111)_, and CMV2b_(18–111)_ (Figure 1B and Supplementary Figure S1).

Typical symptoms were observed in CMV-, CMV2b_(1–76)_-, and CMV2b_(8–111)_-inoculated plants, whereas CMVΔ2b- and CMV2b_(18–111)_-inoculated plants did not develop visible symptoms (Supplementary Figure S1). However, as shown in Figure 1B, RNA gel blot analysis confirmed that the three CMV genomic RNA1, RNA2, and RNA3 transcripts, as well as both subgenomic RNA4 and 4A transcripts, were accumulated in non-inoculated systemic leaves for all chimeric viruses detected using the 3′-untranslated region (3′-UTR) as a probe (Figure 1B). This indicated that the infiltration of the mixture could sustain the replication of both subgenomic RNA4 and 4A. RT-PCR and sequencing analysis of viral RNAs isolated from infected plants confirmed that all chimeric 2bx mutations were genetically stable in *N. benthamiana* (Nb) plants. Similar symptom development and the accumulation of viral RNAs were obtained in the Nb plants inoculated with sap extracted from these each chimeric CMV-infected Nb leaves (Figure 1B).

### Detection of the dsRNA and AGO binding activities of the deletion mutants of the 2b protein

**Figure 2:**
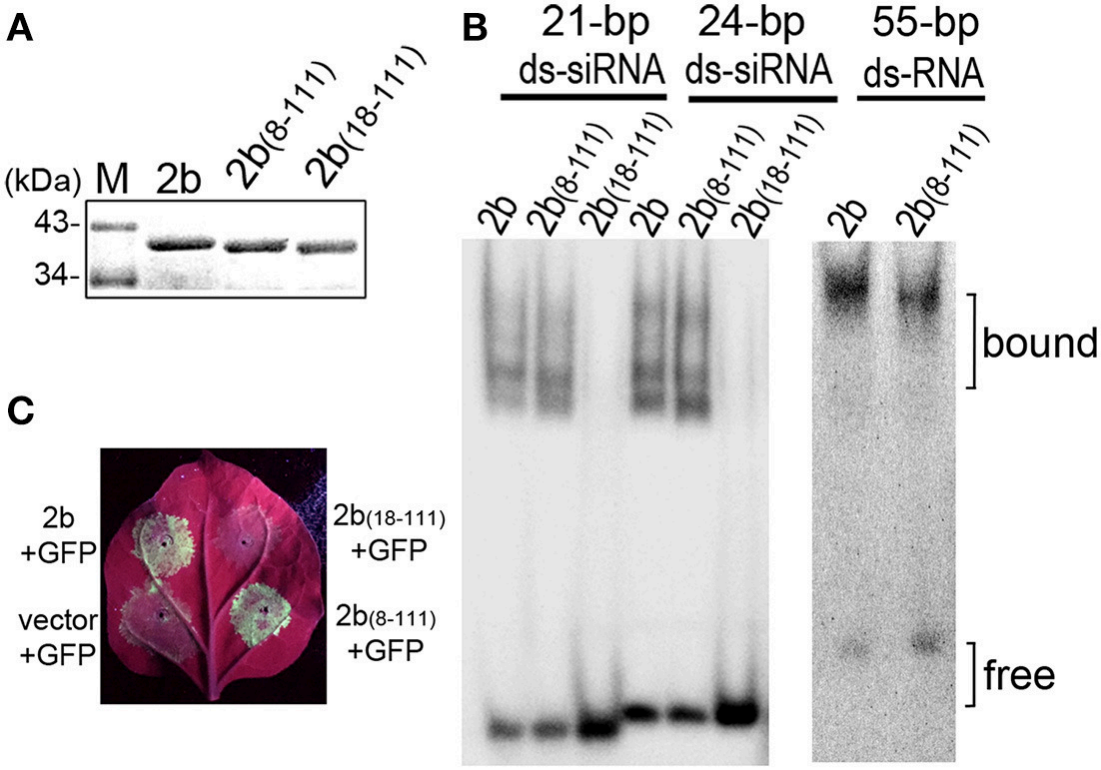
**Detection of the suppression of the transgene-induced silencing and dsRNA-binding activity of 2b and its mutations. (A)** Purified GST-tagged 2b, 2b_(8–111)_, and 2b_(18–111)_. **(B)** Gel mobility shift assays for the detection of the dsRNA binding affinity. GST-tagged 2b, 2b_(8–111)_, and 2b_(18–111)_ were incubated with siRNA duplexes and long dsRNA. 2b_(8–111)_ showed a high affinity for 21/24-nt siRNA duplexes, similar to 2b, but showed a reduced affinity for long dsRNA; 2b_(18–111)_ showed no binding to 21/24-nt siRNA duplexes. **(C)** GFP fluorescence in the leaves of *Nb* plants coinfiltrated with GFP and 2b or mutants. Coinfiltration of GFP with vector was used as control. Photographs were taken under UV light at 4 dpi.

We previously uncoupled the 2b domain requirements for dsRNA binding and nucleolar targeting from the physical interaction with AGO proteins (Duan et al., 2012). The 61 aa N-terminal end [2b_(1–61)_], which contains the complete α1-linker-α2 structure involved in dsRNA binding, retained the wild-type 2b ability to bind 21- and 24-nt siRNA duplexes, whereas 2b_(13–111)_ exhibited weak affinity for 21-nt siRNA but showed no detectable affinity for 24-nt siRNA (Duan et al., 2012). To characterize the dsRNA binding activities of 2b_(8–111)_ and 2b_(18–111)_, which corresponded to the deletion mutants constructed in the context of the CMV RNA2 genome (Figure 1A), we performed electrophoretic mobility shift assays (EMSAs) with the full-length of 2b, 2b_(8–111)_, and 2b_(18–111)_ expressed and purified as GST fusion proteins (Figures 2A,B). Similar to previous results (Duan et al., 2012), 2b exhibited high affinity for either small 21- and 24-nt siRNA duplexes or long dsRNA (Figure 2B). 2b_(8–111)_ was almost as active as 2b in binding to the 21- and 24-nt siRNA duplex but exhibited reduced affinity for long dsRNA (Figure 2B). Deletion of 17 aa from the N terminus abolished both of the siRNA dsRNA binding activities (Figure 2B), which indicated that further deletion of 4 aa (from 13 to 17 aa) in the N-terminal α1 helix completely abolished the weak affinity for 21-nt siRNA of 2b_(13–111)_. Consistently, 2b_(13–111)_ retained the partial silencing suppression activity detected using an *Agrobacterium* coinfiltration assay (Duan et al., 2012); however, 2b_(18–111)_ failed to suppress GFP silencing as indicated by the lack of green fluorescence in the co-infiltrated leaves, in which 2b_(8–111)_ was almost as active as 2b in the suppression of GFP silencing (Figure 2C).

**Figure 3:**
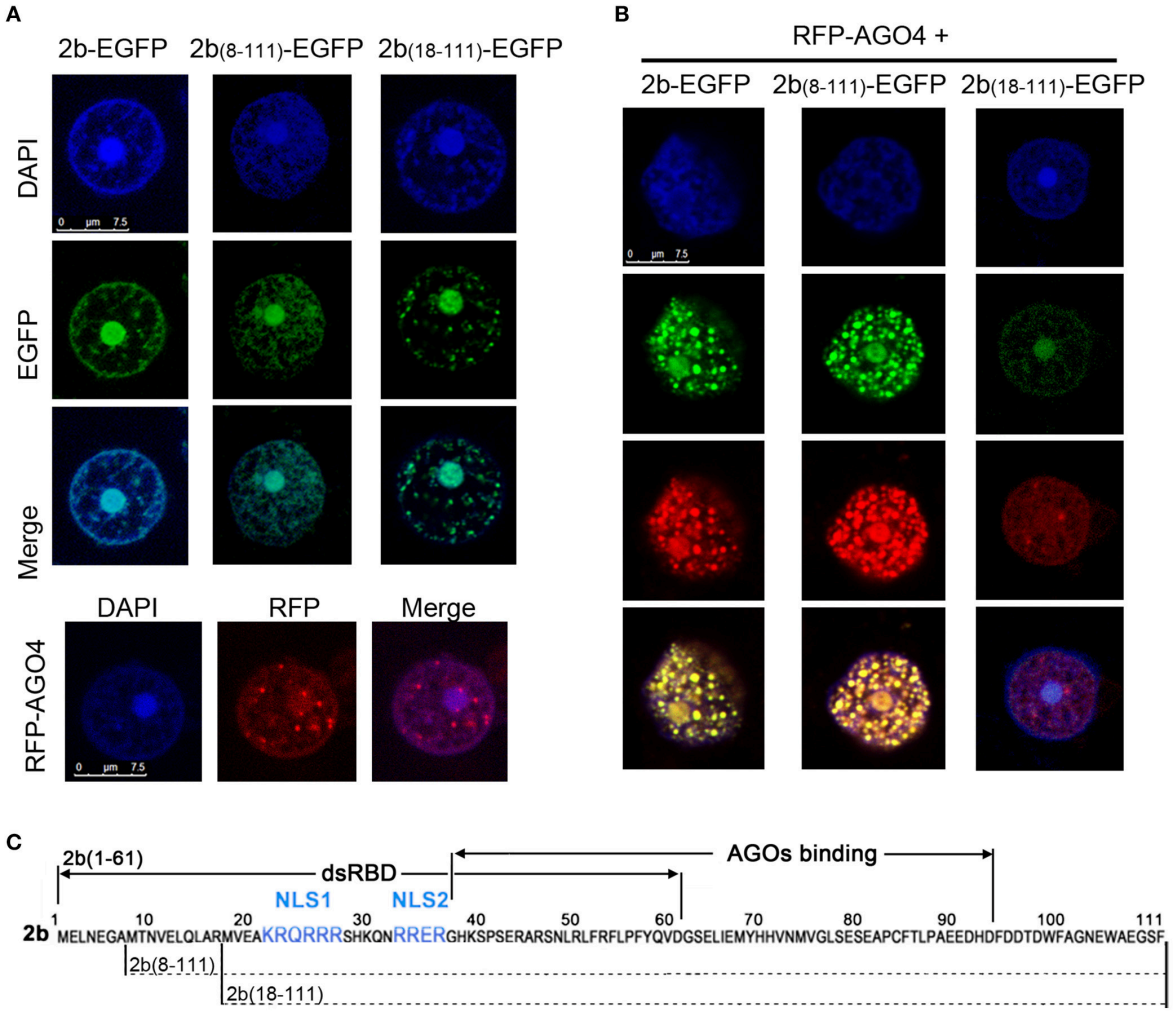
**Colocalization pattern of 2b or its derivative mutants with AGO4 in Nb leaf epidermal cells**. **(A)** Subcellular localization of 2b-EGFP, 2b_(8–111)_-EGFP, 2b_(18–111)_-EGFP, and RFP-AGO4 in *Nb* leaf epidermal cells. DAPI staining was performed to represent the nuclei. Bars = 7.5 μm. **(B)** Subcellular location of the coexpression of 2b-EGFP, 2b_(8–111)_-EGFP, or 2b_(18–111)_-EGFP with RFP-AGO4. **(C)** Diagram of the 2b function domain as previously reported (Duan et al., 2012). NLS, nuclear localization signal.

We next investigated the subcellular localization of 2b_(8–111)_ and 2b_(18–111)_ and their possible interaction with the AGO protein in *N. benthamiana* leaf epidermal cells. 2b_(8–111)_ and 2b_(18–111)_ fused at their C-termini with enhanced green fluorescent protein (EGFP) were transiently expressed in *N. benthamiana* via *Agrobacterium*-mediated infiltration. The 2b-EGFP (Duan et al., 2012) was used as a control. Similar to 2b-EGFP, both 2b_(8–111)_-EGFP, and 2b_(18–111)_-EGFP were mainly detected in the nucleus with dense fluorescence in the nucleoli (Figure 3A), consistent with both deletion mutants containing NoLS from the 13 to 37 region, including both NLSs (Figure 3C). This allowed for the accumulation of the fusion protein in the nucleoli and nucleus (Duan et al., 2012).

Coexpression of 2b-EGFP and RFP-AGO4 resulted in their colocalization in the nucleus with dense fluorescence in nucleus-associated bodies (Figure 3B), which was consistent with our previous finding that the redistribution of 2b-EGFP in the nucleus when coexpressed with AGO proteins (Duan et al., 2012). Similar nuclear colocalization and redistribution was observed for 2b_(8–111)_-EGFP, but not 2b_(18–111)_, when they were coexpressed with RFP-AGO4 (Figure 3B). Intriguingly, the densities of fluorescence were greatly reduced for both 2b_(18–111)_-EGFP and RFP-AGO4 when they were coexpressed compared to that of each when expressed alone (cf. Figures 3A,B). One of the possible explanations might be that the interaction of 2b_(18–111)_-EGFP and RFP-AGO4 caused the instability of both proteins due to 2b_(18–111)_'s lack of binding to ds-siRNA and suppression of silencing (Figures 2B,C). Nevertheless, these data demonstrate that the deletion of 17 aa from the N terminus of the 2b protein completely abolished both the long dsRNA and ds-siRNA binding activity, whereas the deletion of 7 aa from the N terminus retained the wild-type 2b abilities in binding both ds-siRNA and AGO protein but reduced the affinity for binding long dsRNA compared to wild-type 2b.

### Correlation of virulence and viral RNA levels with different 2b mutations in wild-type *Arabidopsis* plants

**Figure 4:**
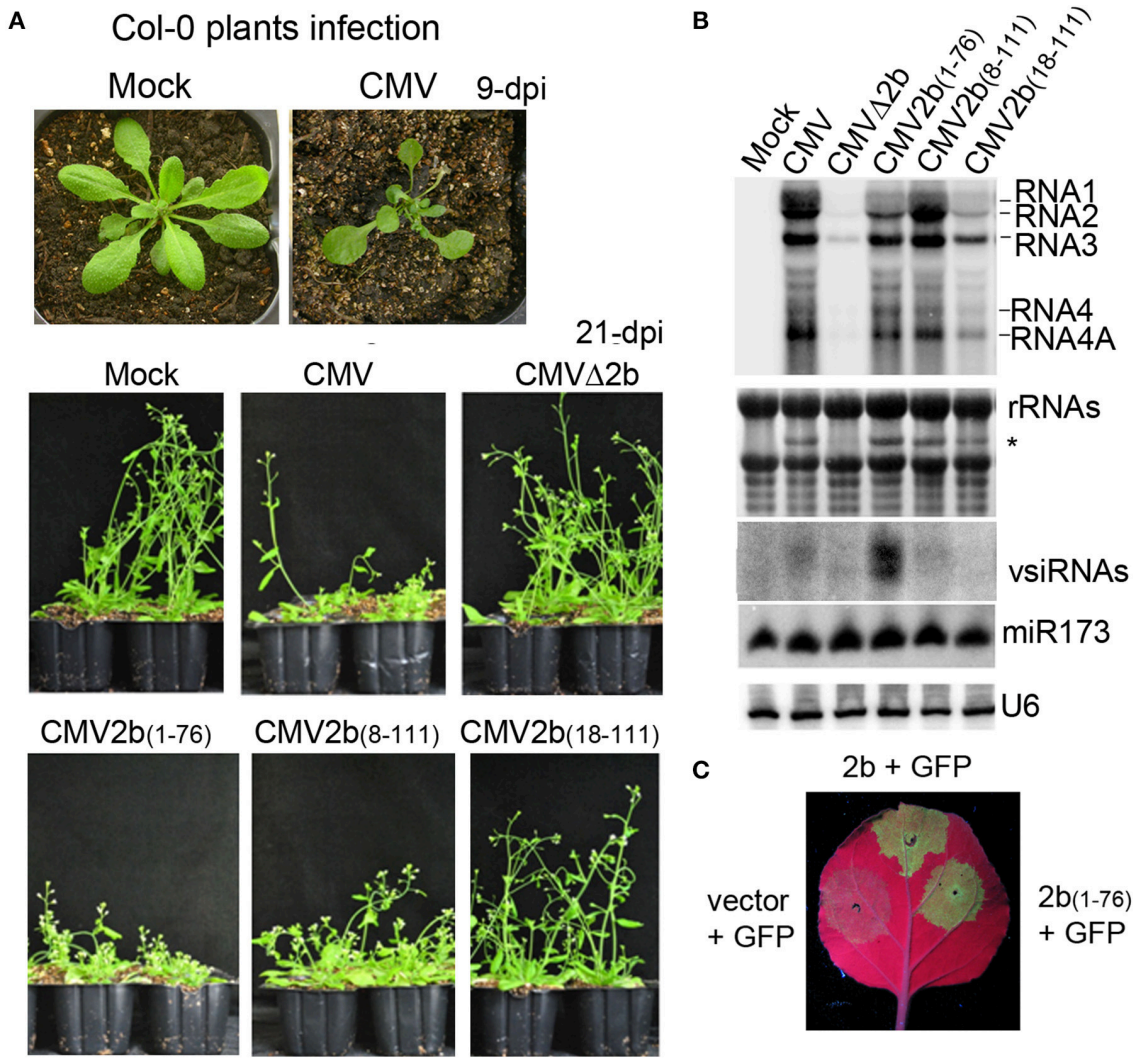
**Effects of 2b and its mutants on the virulence and accumulation of viral RNAs in infection with wild-type ***Arabidopsis*** plants**. **(A)** Disease symptoms in wild-type *Arabidopsis* plants inoculated with CMV and CMV2b_(x)_ at 9 dpi and 21 dpi. **(B)** RNA gel blot detection of viral genomic RNAs and vsiRNAs. Methylene blue-stained ribosome rRNA and U6 were used as the loading control. **(C)** GFP fluorescence in the leaves of *Nb* plants coinfiltrated with GFP and 2b or mutants. Coinfiltration of GFP with vector was used as control.

To investigate the biological functions of the different biochemical properties of the 2b protein in the context of CMV infection in plants, wild-type *Arabidopsis* plants were inoculated with sap extracted from the above *N. benthamiana* leaves infected with CMV, CMVΔ2b, CMV2b_(1–76)_, CMV2b_(8–111)_, or CMV2b_(18–111)_. The development of disease symptoms in *Arabidopsis* plants was monitored. At 9 days post-inoculation (dpi), plants infected with wild-type CMV displayed severe developmental defects, including reduced leaf size and a shortened petiole, and all new leaves were aggregatecd in the center of the plants as observed at 21 dpi (Figure 4A). Plants inoculated with CMVΔ2b displayed no symptoms and normal growth compared to mock infection plants. CMV2b_(1–76)_-infected plants displayed severe developmental defects similar to CMV-infected plants, and the development of both CMV- and CMV2b_(1–76)_-infected plants was arrested with short and defective inflorescence at 21 dpi (Figure 4A). CMV2b_(8–111)_-infected plants also showed typical defects in development and inflorescence, albeit less stunting. However, CMV2b_(18–111)_-infected plants exhibited very mild symptoms, and plant growth was not arrested (Figure 4A). RT-PCR and sequencing analysis of viral RNAs isolated from infected plants confirmed that all chimeric 2bx mutations are genetically stable in *Arabidopsis*.

RNA gel blot analysis confirmed that three CMV genomic (RNA1, RNA2, and RNA3) and both subgenomic (RNA4 and 4A) transcripts were accumulated in the systeimic leaves of these infected plants (Figure 4B). Consistent with the degree of disease, minimal and small quantities of viral RNAs in CMVΔ2b- and CMV2b_(18–111)_-infected plants were detected (Figure 4B). Intriguingly, similar severities of disease symptoms were observed for CMV-, CMV2b_(1–76)_-, and CMV2b_(8–111)_-infected plants; however, the level of viral RNAs in CMV2b_(1–76)_-infected plants was obviously lower than that in CMV- and CMV2b_(8–111)_-infected plants (Figure 4B). 2b_(1–76)_ lacked the AGO binding domain but retained the dsRNA-binding domain and the silencing suppression activity (Figure 4C). Therefore, we examined the production of viral siRNAs (vsiRNAs) in these infected plants. High levels of vsiRNAs were detected in CMV2b_(1–76)_-infected plants compared to wild-type and those infected with other chimeric viruses (Figure 4B), which was consistent with the low level of viral RNAs in these plants. No major differences in the accumulation of miR173 were detected following infection with either wild-type or each mutant CMV, which supported an earlier observation that CMV infection does not alter miRNA accumulation (Diaz-Pendon et al., 2007). These results demonstrate that N terminal dsRNA binding activity is responsible for the induction of the virulence of CMV, which does not necessarily correlate with the accumulation of viral RNAs, and 2b-AGO binding is likely required for CMV to suppress the silencing of viral RNAs in *Arabidopsis* plants.

### Correlation of virulence and viral RNA levels with different 2b mutations in RDR1/RDR6-deficient mutants

**Figure 5:**
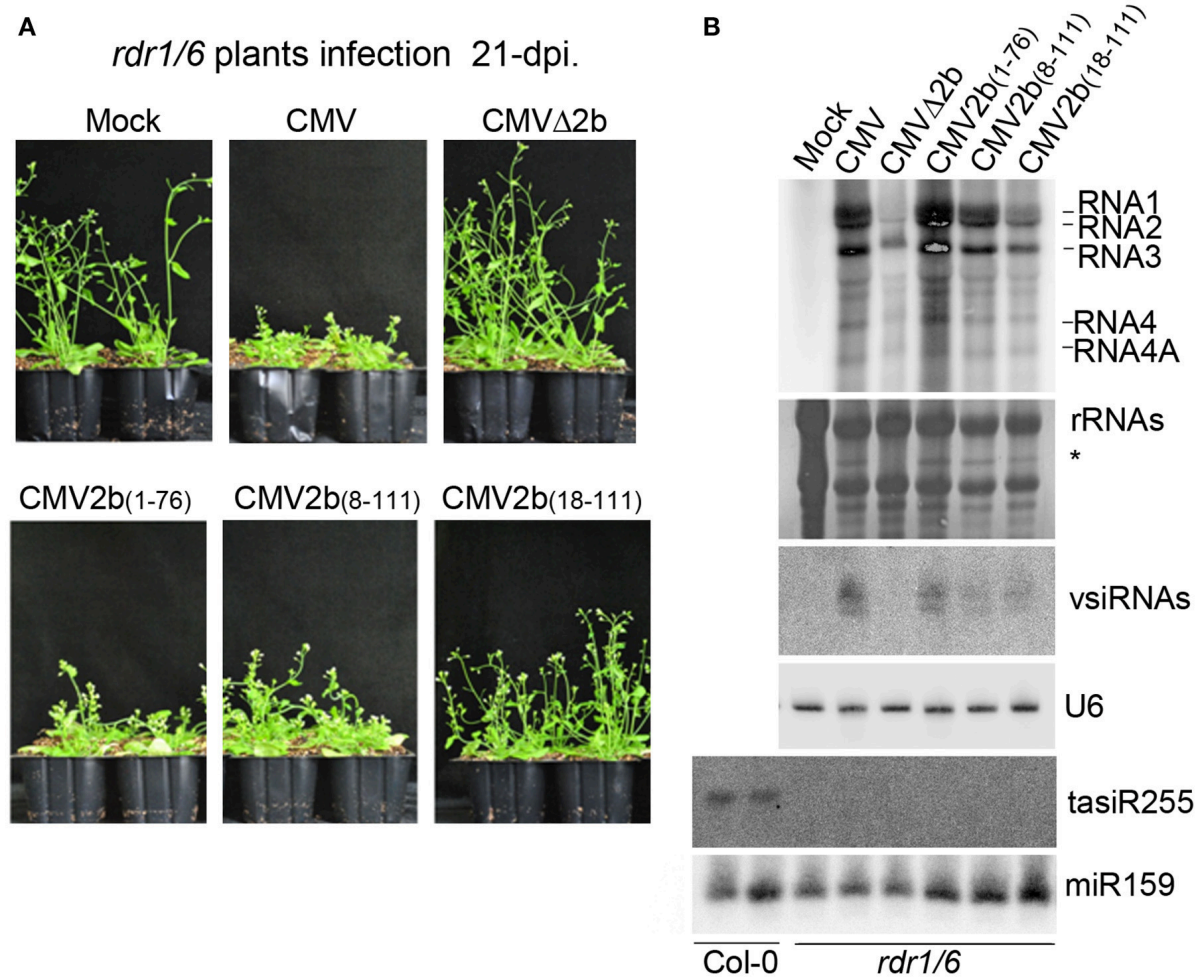
**Effects of 2b and its mutants on the virulence and accumulation of viral RNAs in infection of ***rdr1/6*** plants**. **(A)** Disease symptoms in *rdr1/6* plants inoculated with CMV and CMV2b_(*x*)_ at 21 dpi. **(B)** RNA gel blot detection of viral genomic RNAs and vsiRNAs. tasiR255 was used to verify the *RDR* mutant alleles. Methylene blue-stained ribosome rRNA and U6 as well as miR159 were used as the loading controls.

Previous studies showed that the 2b gene of the CMV Fny strain is required for interference with RDR1- and RDR6-dependent antiviral silencing (Diaz-Pendon et al., 2007; Dong et al., 2016). To examine whether the accumulation of CMV-derived vsiRNAs in CMV2b_(1–76)_-infected plants was dependent on RDR proteins, we inoculated *Arabidopsis* with double mutants of RDRs (RDR1 and RDR6) with wild-type CMV, CMVΔ2b, CMV2b_(1–76)_, CMV2b_(8–111)_, or CMV2b_(18–111)_. *Trans-acting* siRNA tasiR255 was absent in the tested *rdr1/6* plants, verifying that the *RDR* mutant alleles (Figure 5B). CMVΔ2b remained defective in inducing virulence in *rdr1/6* mutant; CMVΔ2b-infected plants displayed no symptoms and normal growth compared to mock infection plants (Figure 5A). Similar to infected wild-type *Arabidopsis* plants, *rdr1/6* plants infected with CMV-, CMV2b_(1–76)_-, and CMV2b_(8–111)_ displayed severe developmental defects, and whole plant development was arrested, although less stunting was observed in CMV2b_(8–111)_-infected *rdr1/6* plants (Figure 5A). CMV2b_(18–111)_-infected *rdr1/6* mutant plants exhibited mild but clear disease symptoms (Figure 5A). The inflorescences of CMV2b_(18–111)_-infected *rdr1/6* plants were shorter and defective compared to those of CMV2b_(18–111)_-infected wild-type plants (Figure 4A), which demonstrated that 2b_(18–111)_ retained partial activity in the suppression of RDR6- and/or RDR1-mediated antiviral defense.

We further conducted RNA gel blot hybridizations to compare the accumulation of viral RNAs in these infected *rdr1/6* mutant plants. Consistent with the asymptomatic phenotype, minimal levels of viral RNAs in CMVΔ2b-infected plants were detected (Figure 5B). Interestingly, a similar severity of disease symptoms was observed for CMV-, CMV2b_(1–76)_-, and CMV2b_(8–111)_-infected *rdr1/6* plants; however, the accumulation level of viral RNAs in CMV2b_(1–76)_-infected plants was clearly higher than that in CMV- and CMV2b_(8–111)_-infected mutant plants (Figure 5B), opposite of that detected in infected wild-type *Arabidopsis* plants, in which a lower level of viral RNAs accumulated in CMV2b_(1–76)_-infected plants than in CMV- and CMV2b_(8–111)_-infected plants (Figure 4B).

We then examined the production of vsiRNAs. Remarkably, vsiRNAs in CMV2b_(1–76)_-infected *rdr1/6* plants (Figure 5B) were greatly reduced compared to CMV2b_(1–76)_-infected wild-type Col-0 plants (Figure 4B). These results indicated that RDR6 and/or RDR1 play role(s) in partially silencing CMV RNAs disrupted by 2b-AGO binding activity. Taking into account of the low level of viral RNAs and the high level of vsiRNAs in CMV2b_(1–76)_ infected wild-type plants, our data demonstrate that the 2b-dsRNA binding activity is insufficient to suppress the host degradation of viral RNA that requires the functions of RDR6 and/or RDR1.

## Discussion

In our previous study, we characterized the SD-CMV 2b protein in terms of subcellular localization, RNA binding, AGO interaction, and the suppression of RNA silencing (Duan et al., 2012). We found that dsRNA sequestration is the predominant mechanism by which 2b suppresses silencing and that the 2b-AGO interaction is not essential for its suppressor function. In this study, we further explored the biological significance of different functional activities of the 2b protein in the context of virus infection. By creating mutants of SD-CMV that expressed different 2b functional domains, either retaining the dsRNA-binding activity or the AGO-binding activity, we found that 2b's dsRNA-binding activity was essential for virulence and viral RNA propagation, whereas the 2b-AGO interaction was necessary for interference with RDR-dependent antiviral silencing in *Arabidopsis*.

The 2b_(8–111)_ mutant protein with 7 aa deleted from the N terminus was almost as active as wild-type 2b in binding to 21- and 24-nt ds-siRNA duplex and in the suppression of transgene *GFP* silencing (Figure 2). Therefore, CMV2b_(8–111)_ caused wild-type CMV-like to impact both the viral RNA level and the virulence in either wild-type *Arabidopsis* or *rdr1/6* mutant plants. However, we also noted that the stunted phenotype was less severe in CMV2b_(8–111)_-infected plants. This might be related to its reduced activity in binding long dsRNA compared to that of wild-type 2b (Figure 2B). This observation suggests that the first 7 aa of the 2b protein might affect the N-terminal α1 helix, which is followed by the short linker and the α2 helix structure involved in long dsRNA binding (Chen et al., 2008). Indeed, we previously found that 2b_(13–111)_ with the deletion of the N-terminal 12 aa was defective in binding long dsRNA and 24-nt ds-siRNA but retained very weak 21-nt ds-siRNA binding ability, which revealed that the N-terminal α1 helix is essential for binding to long dsRNA (Duan et al., 2012). Thus, even 2b_(8–111)_ was as active as wild-type 2b in binding both 21- and 24-nt ds-siRNA, it was defective in binding long dsRNA. Thus, 2b_(8–111)_ may be defective in binding, for example, endogenous long non-coding RNAs that have emerged as new regulatory elements with essential roles in plant development and stress signaling pathways (Wu et al., 2013; Zhu et al., 2014; Wang et al., 2015), which may explain the less stunted phenotype in CMV2b_(8–111)_-infected plants.

Deletion of 17 aa from the 2b N terminus abolished both the siRNA-binding and silencing suppressor activities (Figure 2). CMV2b_(18–111)_ infection never achieved the wild-type CMV level of viral RNAs in either wild-type *Arabidopsis* or *rdr1/6* mutant plants (Figures 4, 5). However, in comparison with CMVΔ2b infection, the level of viral RNAs was clearly higher in CMV2b_(18–111)_-infected wild-type *Arabidopsis*, but not obvious in *rdr1/6* plants, which implied that the 2b_(18–111)_ had a role in countering the RDR-dependent defense against CMV accumulation. We previously found that *in vivo*, the 2b-AGO interaction depends also on the nucleolar targeting of the 2b protein (Duan et al., 2012). 2b_(18–111)_ retained the AGO-binding domain, and its nucleolar targeting was evident (Figure 3A). However, taking into account that the instability of both the 2b_(18–111)_–EGFP and RFP-AGO4 fusion proteins when they were co-expressed (Figure 3B), we speculated that it would decrease the effect of 2b_(18–111)_ on countering RDR-dependent resistance in the absence of dsRNA binding activity. A previous study that examined the 2b-AGO4 interaction using the BiFC assay found that the fluorescent signal of the 2b-AGO4 interaction was reduced in *rdr2* mutant plants, which compromised the accumulation of 24-nt siRNAs (Hamera et al., 2012). Taken together, these findings suggest that a lack of siRNAs in the formation of the 2b_(18–111)_-AGO4 complex might result in the degradation of both proteins.

The effect of the 2b-AGO interaction in counteracting RDR-dependent antiviral silencing was substantiated by comparing the levels of viral RNAs and siRNAs in CMV2b_(1–76)_-infected wild-type *Arabidopsis* and *rdr1/6* mutant plants (Figures 4, 5). Retaining the dsRNA-binding activity but lacking the AGO-binding domain, 2b_(1–76)_ failed to suppress the RDR-dependent degradation of viral RNAs, resulting in the production of a large quantity of vsiRNAs and a decrease in the viral RNA level (Figure 4B). This finding clearly demonstrated that the 2b-AGO binding activity is required for CMV to counter the host's RDR-dependent degradation of viral RNAs. The physical interaction of 2b with AGOs requires the region encompassing residues 62 to 94 (Duan et al., 2012). Although this region is highly variable in sequence, it is present among all of the cucumoviral 2b proteins (Ding et al., 1994), which reveal its important in *vivo* function for CMV infection.

We found that the 2b dsRNA binding activity is responsible for the induction of virulence, which did not necessarily correlate with the level of CMV RNAs (Figure 4). This is consistent with a previous finding that Fny-CMV2bNLS, an Fny strain expressing the 2b mutant with an additional NLS and enhanced nuclear targeting, increased viral virulence but decreased virus accumulation and increased vsiRNAs (Du et al., 2014a). The authors therefore proposed that partitioning the 2b protein between the cytoplasmic and nuclear/nucleolar compartments allows CMV to regulate the balance between virus accumulation and damage to the host (Du et al., 2014a). We previously found that the 2b-AGO interaction redistributed both the 2b and AGO proteins in the nucleus (Duan et al., 2012). The nucleus/nucleolus-localized 2bNLS failed to increase virus accumulation in Fny-CMV2bNLS infection, which might be attributed to the disrupted redistribution of 2b-AGO. This likely resembled the 2b_(1–76)_ failure in interactions with AGO proteins and the inhibition of viral RNA degradation in Fny-CMV2bNLS infection. It was also reported that Fny-CMV2blm expressing the 2b mutant defective in ds-siRNA binding activity drastically attenuated the virulence in wild-type *Arabidopsis* plants (Dong et al., 2016). Virulence could be efficiently rescued in CMV2blm-infected plants harboring RDR6-deficient mutations, including *rdr6, rdr1/6, rdr2/6*, and *rdr1/2/6*, but not *rdr1* and *rdr2* mutant plants. Viral RNAs also accumulated to higher levels in *rdr6* and *rdr1/6* than in wild-type *Arabidopsis* plants (Dong et al., 2016). Unlike the wild-type N terminus of 2b, which was required to form dimers, tetramers and oligomers, 2blm could only form dimers (Dong et al., 2016). Therefore, the rescued virulence and viral RNA level in CMV2blm-infected RDR6-deficient mutant plants was suggested due to the low oligomerization of the 2blm that directly weakened ds-siRNA binding activity (Dong et al., 2016). The 2blm contained the full-length sequence with double alanine substitution (L15A and M18A) mutations at the N-terminus but retained the long dsRNA binding domain (Dong et al., 2016). Thus, unlike 2b_(18–111)_, which caused the instability of the interacting 2b_(18–111)_-AGO proteins (Figure 3B), 2blm might bind to AGO but retain the stability of two proteins due to its long dsRNA binding activity. This may rescue the virulence in *rdr6* mutant plants, in which the RDR1-mediated degradation of viral RNAs might be suppressed by the 2blm-AGO interaction. In our study, the N-terminal 17 aa deletion mutant 2b_(18–111)_ contained the AGO-binding domain but was defective in binding long and short dsRNA. Neither severe disease symptoms nor high viral RNA level were obtained with CMV2b_(18–111)_ infection in *rdr1/6* plants (Figure 5), which demonstrated the failure of the *in vivo* 2b_(18–111)_-AGO-dependent suppression of the host degradation of CMV RNAs in the absence of 2b dsRNA binding activity.

In summary, although the silencing suppression activity of 2b relies on its dsRNA binding capacity and is independent of its interaction with AGO (Duan et al., 2012), we found that in the context of virus infection, the 2b-AGO interaction was indispensable for interference with RDR-dependent antiviral silencing in *Arabidopsis*, and the effect was remarkable in the presence of the 2b dsRNA-binding activity. The 2b dsRNA-binding activity was essential for virulence, probably being related to its effect on the alteration of miR159-regulated transcript levels (Du et al., 2014b). However, in agreement with the dual edge of VSR in the virus-host interactions (Zhao et al., 2016), the 2b protein exerted a precise effect on the regulation of balance between virus accumulation and virulence-induced damage to the host. Binding to AGO proteins might weaken the nucleus/nucleolus localization of 2b and inhibit the RDR-dependent degradation of viral RNAs in the cytoplasm, presumably to maximize the benefit for the virus. The multiple biochemical properties of the 2b protein exerted essential roles in diverse silencing suppressor activities, which cooperatively or independently contributed to the accumulation and virulence of viral RNAs.

## Author contributions

HG, CD, and YF conceived the study and designed the research. YF and JZ performed molecular work. YF, JZ, and SL performed the sampling and analyzed the data. SW assisted construction and infection. HG, YF, and JZ wrote the manuscript and discussed the results and all the authors commented on the manuscript.

## Funding

This study was supported by the Ministry of Science and Technology (2014CB138402) and the Natural Science Foundation of China (No.91519327).

### Conflict of interest statement

The authors declare that the research was conducted in the absence of any commercial or financial relationships that could be construed as a potential conflict of interest.
