# Supplementary material for: CMV2b-AGO Interaction Is Required for the Suppression of RDR-Dependent Antiviral Silencing in Arabidopsis
Source: Front Microbiol. 2016 Aug 24;7:1329. doi: 10.3389/fmicb.2016.01329 (PMC4995204; doi:10.3389/fmicb.2016.01329)
Supplement: Supplementary file 1 [file Table1.PDF]

Table S1 List of primers and DNA/RNA oligos used in this study

name	sequence（5’ to 3’)	Purpose
R2-2b77TGAF	CATGTGAACATGGTGGGATAGTCCGAGTCTGAGGCC	R2a2b(1-76)
R2-2b77TGAR	GGCCTCAGACTCGGACTATCCCACCATGTTCACATG	
R2-2b8-111F	GCAACGAAAGAAGAAAGACGGAATTGAACGAAGGCGC	R2a2b(8-111)
R2-2b8-111R	GCGCCTTCGTTCAATTCCGTCTTTCTTCTTTCGTTGC	
R2-2b18-111F	GAATTGAACGAAGGCGCAACGACAAACGTCGAACTC	R2a2b(18-111)
R2-2b18-111R	GAGTTCGACGTTTGTCGTTGCGCCTTCGTTCAATTC	
GST2b8-111F	GGTTCCGCGTGGATCCATGACAAACGTCGAACTCC	pGEX-4T-2-SD2b-8-111
GST2b8-111R	GGAGTTCGACGTTTGTCATGGATCCACGCGGAACC	
GST2b18-111F	GGTTCCGCGTGGATCCATGGTGGAGGCGAAGAGGC	pGEX-4T-2-SD2b-18-111
GST2b18-111R	GCCTCTTCGCCTCCACCATGGATCCACGCGGAACC	
2b8-111F	TCTAGAGGATCCATGACAAACGTCGAAC	pBI121-35S-SD2b8-111
2bR	GAGCTCCTCGAGTCAGAACGACCCTTCCGC	
2B18-111F	TCTAGAGGATCCATGGTGGAGGCGAAGAGGC	pBI121-35S-SD2b18-111
2bR	GAGCTCCTCGAGTCAGAACGACCCTTCCGC	
2B1-76F	TCTAGAGGATCCATGGAATTGAACGAAGGC	pBI121-35S-SD1-76
2B1-76R	GAGCTCCTCGAGTCATCCCACCATGTTCACATGGTG	
2B18-111-EGFPF	CGAACTCCGGGGGACTCTAGATGGTGGAGGCGAAGAGGC	pBI121-35S-SD2b18-111-EGFP
2B18-111-EGFPR	GCCTCTTCGCCTCCACCATCTAGAGTCCCCCGGAGTTCG	
21bp dsRNA	UCAACAGGAUCGAGCUUAAGG3’	EMSA
	3’GCAGUUGUCCUAGCUCGAAUU5’	
24bp dsRNA	5’UGAAUUUCUGGUAUGGGUCCCGCC3’	
	3’CUACUUAAAGACCAUACCCAGGGC5’	

55bp dsRNA	5’UACAAGACACGUGCUGAAGUCAAGUUUGAGGGA GACACCCUCGUCAACAGGAUCG3’<br><br>3’UGAUGUUCUGUGCACGACUUCAGUUCAAACUCCCUCUGUGGGAGCAG UUGUCCUA 5’	
SD-CMV-R3 1-49F	GTAATCTTACCACTGTGTGTGTGCGTGTGTGTGTGTCGCGTCGTGTC	vsiRNAs blot
SD-CMV-R3 267-306F	CTGATAACGCAATTTCAGTCCGGCCCCTCGTTCCCGAAGT	
SD-CMV-R3 767-806F	GCCGTCGCTCGCCTGTTGAAGTCGCAATTGAACAACATAG	
SD-CMV-R3 1013-1053F	CTATGTTTGCGGACGGAGCCTCACCGGTACTGGTTTATCA	
miR173	GTGATTTCTCTCTGCAAGCGAA	blot
miR159	TAGAGCTCCCTTCAATCCAAA	
tasiR255	TTCTAAGTCCAACATAGCGTACCTGTCTC	
U6	GCTAATCTTCTCTGTATCGTTCC	
