# Supplementary material for: CMV2b-AGO Interaction Is Required for the Suppression of RDR-Dependent Antiviral Silencing in Arabidopsis
Source: Front Microbiol. 2016 Aug 24;7:1329. doi: 10.3389/fmicb.2016.01329 (PMC4995204; doi:10.3389/fmicb.2016.01329)
Supplement: Supplementary file 2 [file Image1.PDF]

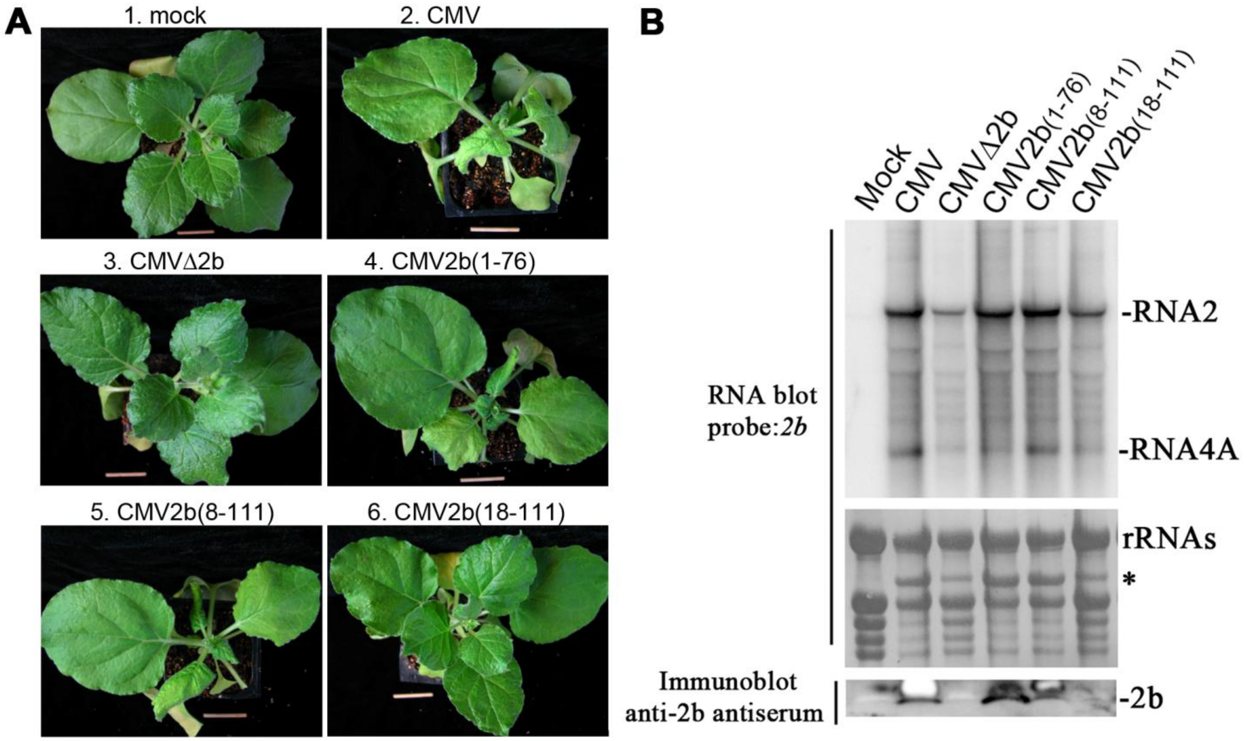

Supplementary Fig. S1 Detection of biological activities of 2b mutants in the context of CMV infection. (A) Disease symptoms on *Nicotiana benthamiana* inoculated with wild-type CMV or chimeric CMV ($CMV2b_{(x)}$), which contains the indicated chimeric RNA2 mutant. Bar=1.5 cm. Diagram of CMV infectious clone construction is present in Figure 1A in the main text. (B) RNA gel blot and immunoblot detection of CMV RNA2 and subgenomic 4A as well as the 2b and its mutant proteins. SD-CMV genomic RNA2 was used as a probe. Methylene blue-stained ribosomal rRNA was used as loading control. * a stained viral RNA used as an indicator of SD-CMV infection. anti-2b antiserum (1:1000) and goat anti-rabbit immunoglobulin G (lgG) secondary antibody were used for immunoblot detection of 2b and mutant proteins.
